# Disseminated fungal infection with *Saprochaete capitata* in acute myeloid leukemia patient: a case report from a developing country

**DOI:** 10.1093/omcr/omae176

**Published:** 2025-01-18

**Authors:** Mamoun W Swaileh, Ayman Dawoud, Razan Malhis, Banan M Aiesh

**Affiliations:** Department of Internal Medicine, An-Najah National University Hospital, Nablus 44839, West Bank, Palestine; Department of Microbiology, An-Najah National University Hospital, Nablus 44839, West Bank, Palestine; Hematology and Oncology Department, An-Najah National University Hospital, Nablus 44839, West Bank, Palestine; Infection Control Department, An-Najah National University Hospital, Nablus 44839, West Bank, Palestine

**Keywords:** *Saprochaete capitata*, invasive fungal infection, acute myeloid leukemia, antifungals, resistance, fungemia

## Abstract

Background: *Saprochaete capitata* may cause fatal infections in immunocompromised patients. This is the first case report of invasive *Saprochaete capitate* infection at an academic-tertiary care center in Palestine. Case presentation: We report a 36—year—old woman who presented with fever and severe neutropenia and was found to have AML/Non M3. While receiving her initial chemotherapy treatment, she encountered a rare fungal infection (*Saprochaete capitata*) that spread throughout her lungs, stomach, spleen, liver, and kidneys, presenting difficulties in both diagnosing and treatment. In addition to being treated with both voriconazole and amphotericin B, the patient underwent surgery to remove the infection source, resulting in a cure. Conclusion: In immunocompromised patients, *Saprochaete *capitata** infection is frequently overlooked. It is essential to give antifungal combinations and to control the source of infection to enhance the outcome for patients.

## Introduction

The frequency of invasive fungal infections (IFIs) caused by opportunistic fungi has recently increased, particularly among patients with hematological malignancies. *Saprochaete capitata*, previously known as *Geotrichum capitatum*, can be commonly found in the environment and dairy products. Invasive infections with *Saprochaete capit*ata have been associated with mortality rates ranging from 57% to 90% in patients diagnosed with acute myeloid leukemia (AML) [1].

Current diagnostic methods are inadequate to investigate the complex epidemiology of IFIs in immunocompromised patients. Diagnosing invasive *Saprochaete* infections is challenging since they need direct isolation from blood or tissue samples. Accurate identification based on phenotypic characteristics is difficult because it is closely related to other fungi [2]. In this report, we present the first case of invasive *Saprochaete capit*ata infection in a patient with AML treated medically and surgically at a tertiary care center in the West Bank, Palestine.

## Case report

A 36-year-old woman with no medical history was admitted to An-Najah National University Hospital in the West Bank, Palestine for fever and pancytopenia without a known source of infection*.* The lab findings indicated a neutrophil count of 300 cells per microliter and she received a diagnosis of non-M3 AML. On the eighth day of chemotherapy, she experienced neutropenic fever and received IV antibiotics following culture collection. One week later, her fever persisted and the antibiotics were switched to meropenem and vancomycin.

On the eighth day of neutropenic fever, a pan-computed tomography (CT) scan revealed bibasal pulmonary glass opacity, an enlarged spleen measuring 15 cm with several hypodense lesions*.* Because of inadequate vascular access, a catheter was placed in the right internal jugular vein. It was believed that this overall picture, combined with the positive galactomann test, indicated a widespread fungal infection, leading to the initiation of oral voriconazole. Severe thrombocytopenia prevented a biopsy from being performed, leading to an unclear diagnosis. Furthermore, there were no abnormalities detected during the ophthalmological examination and transthoracic echocardiogram.

**Figure 1:**
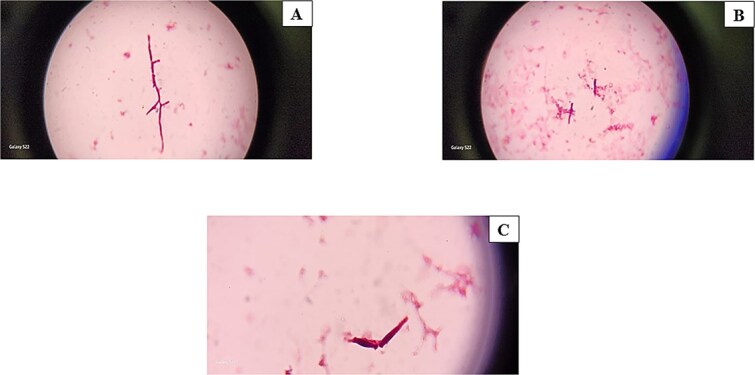
**(A-C):** Gram-stained smear from blood showing short and long-branched filamentous yeast cells.

**Figure 2:**
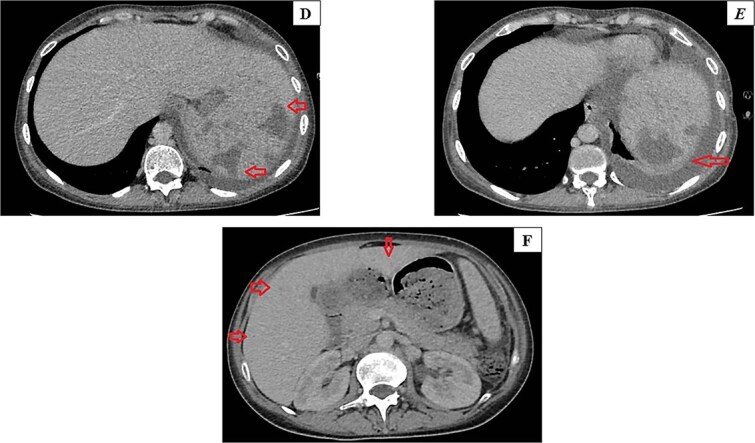
**(D-F):** Computed tomography (CT) scan showing hypodense lesions in spleen and liver.

On the ninth day of treatment, primary fungal growth was observed in the peripheral blood culture, leading to the initiation of a broader antifungal agent (IV caspofungin) (Fig. 1). Nonetheless, the final growth revealed growth of *Saprochaete capitata;* a fungi that exhibited varying appearances when grown on blood and chocolate agar, with dry white colonies on blood agar and cotton colonies with a frosted glass appearance on chocolate agar. Based on the susceptibility report, treatment started with amphotericin B (minimum inhibitory concentrations were: voriconazole < 2 mg/l, amphotericin B < 0.25 mg/l and caspofungin > 32 mg/l), leading to a decrease in inflammatory markers, fever frequency, and cell recovery count. Subsequent blood cultures revealed no signs of bacterial growth. A second bone marrow biopsy revealed no malignant cells, and she was prescribed voriconazole tablets for a six-week course upon leaving the hospital. She was admitted again for consolidation chemotherapy but experienced neutropenic fever. Even with antibiotics and voriconazole, her fever persisted, and the CT scan indicated the disappearance of the lung opacity, but revealed that the splenic hypodense lesions had grown in both size and number (4.5 *×* 5 cm, previously only subcentimeters). Furthermore, there were newly emerged small hypodense lesions in both kidneys, as well as numerous hypodense lesions in the liver (Fig. 2)*.* Amphotericin B IV was administered in combination with oral voriconazole, and a spleen biopsy guided by CT was conducted, however, the culture revealed no organism growth*.* Following a two-week course of dual antifungal treatment, her condition improved; however, she had to be readmitted due to the recurrence of fever. Despite receiving antifungals and steroids, no improvement was seen in her condition, with the spleen having the largest lesions. She had surgery involving resection of spleen removal, part of the stomach, and part of the pancreas due to extensive adhesions and to control source of infection. The histopathological analysis revealed fungal spores and septate hyphae branching at sharp angles, while the tissue culture did not indicate any growth. Voriconazole and amphotericin B were prescribed for another three weeks, and a CT scan showed marked improvement in the hypodense liver and kidney lesions*.*

## Discussion

This patient had various factors associated with IFIs, like profound neutropenia, broad-spectrum antibiotics, chemotherapy, and central vascular catheterization. At present, the most widely utilized way to diagnose fungemia is by blood culture, which determines the pathogen’s species level, and performs susceptibility testing. Direct microscopy utilizing Gram stain is strongly recommended to decrease culture turnaround time [3]. Delay in identifying the problem may occur because symptoms similarity with other IFIs [1, 4]. Her test for Aspergillus galactomann was positive, resembling that of two cases of *Saprochaete capitata* bloodstream infections in leukemia patients, likely due to cross-reactivity with Aspergillus antigen [5].

The recommended treatment approach for IFIs caused by *Saprochaete capitata* relies on expert opinions and case reports, suggesting amphotericin B with or without flucytosine as the first-line therapy. In addition to identifying *Saprochaete* isolates, performing susceptibility testing is difficult because there are no clinically established breakpoints. In our study, the lowest MIC values for *Saprochaete capitate* were seen with amphotericin B (<0.25 mg/l) and voriconazole (< 2 mg/l), as opposed to the lowest MIC values reported for voriconazole (0.125 mg/l) and amphotericin B (1 mg/l) [6]. Our results confirmed earlier research showing decreased susceptibility to fluconazole [7, 8]. Successful treatment using amphotericin B was reported, either alone or together with voriconazole [1, 7]. Caspofungin had the highest MIC as seen in previous cases, therefore it is advisable to refrain from using it as empirical treatment [7].

Antifungal treatment and managing the source of infection were necessary in treating invasive *Saprochaete spp*. As a result, surgical procedures for early source control are deemed essential for both diagnosis and treatment, as it is vital to obtain tissue culture to properly direct therapy [9, 10] Following the surgical procedure, the patient showed ongoing progress until complete recovery through the use of amphotericin B and voriconazole. At present, the patient is progressing satisfactorily during monthly check-ups. Antifungal treatment and source control are crucial for treating invasive *Saprochaete* infections, requiring surgery and tissue culture collection for proper diagnosis and treatment.

## Consent

Written informed consent for publication from the patient to publish this case report and accompanying images was obtained. A copy of the written consent is available for review by the journal’s editor.

## Guarantor

Dr. Banan M. Aiesh.
