# Correction to: Disseminated fungal infection with *Saprochaete capitata* in acute myeloid leukemia patient: a case report from a developing country

**DOI:** 10.1093/omcr/omaf221

**Published:** 2025-09-28

This is a correction to: Mamoun W Swaileh, Ayman Dawoud, Razan Malhis, Banan M Aiesh, Disseminated fungal infection with *Saprochaete capitata* in acute myeloid leukemia patient: a case report from a developing country, *Oxford Medical Case Reports*, Volume 2025, Issue 1, January 2025, omae176, https://doi.org/10.1093/omcr/omae176

The originally published version of this manuscript contained an error in the first author's name.

This has been corrected.

